# Pirfenidone inhibits TGF‐β1‐induced metabolic reprogramming during epithelial‐mesenchymal transition in non‐small cell lung cancer

**DOI:** 10.1111/jcmm.18059

**Published:** 2023-12-23

**Authors:** Shuling Zhang, Yuanmei Wang, Daiqin Luo, Zhimei Cheng, Qibing Zeng, Guoze Wang, Mengxue Chen, Shuai Zhang, Peng Luo

**Affiliations:** ^1^ Guizhou Medical University Guiyang China; ^2^ Key Laboratory of Environmental Pollution Monitoring and Disease Control, Ministry of Education School of Public Health Guiyang China; ^3^ State Key Laboratory of Functions and Applications of Medicinal Plants Guizhou Medical University Guiyang China; ^4^ Guizhou Provincial Engineering Research Center of Food Nutrition and Health Guizhou Medical University Guiyang China

**Keywords:** EMT, glycolysis, NSCLC, pirfenidone, TGF‐β1

## Abstract

Metastasis is an important contributor to increased mortality rates in non‐small cell lung cancer (NSCLC). The TGF‐β signalling pathway plays a crucial role in facilitating tumour metastasis through epithelial‐mesenchymal transition (EMT). Glycolysis, a key metabolic process, is strongly correlated with NSCLC metastasis. Pirfenidone (PFD) has been shown to safely and effectively inhibit TGF‐β1 in patients with lung diseases. Furthermore, TGF‐β1 and glycolysis demonstrate an interdependent relationship within the tumour microenvironment. Our previous study demonstrated that PFD effectively inhibited glycolysis in NSCLC cells, prompting further investigation into its potential antitumour effects in this context. Therefore, the present study aims to investigate the potential antitumour effect of PFD in NSCLC and explore the relationship among TGF‐β1, glycolysis and EMT through further experimentation. The antitumour effects of PFD were evaluated using five different NSCLC cell lines and a xenograft tumour model. Notably, PFD demonstrated a significant antitumour effect specifically in highly glycolytic H1299 cells. To elucidate the underlying mechanism, we compared the efficacy of PFD after pretreatment with either TGF‐β1 or a TGF‐β receptor inhibitor (LY2109761). The energy metabolomics analysis of tumour tissue demonstrated that PFD, a chemosensitizing agent, reduced lactate and ATP production, thereby inhibiting glycolysis and exerting synergistic antineoplastic effects. Additionally, PFD combined with cisplatin targeted TGF‐β1 to inhibit glycolysis during EMT and enhanced the chemosensitization of A549 and H1299 cells. The magnitude of the anticancer effect exhibited by PFD was intricately linked to its metabolic properties.

## 1 INTRODUCTION

Lung cancer has the second highest incidence and the highest mortality among cancers worldwide^1^ and has become a serious public health problem because of its high morbidity and mortality. According to its pathological classification, lung cancer is divided into two types: non‐small cell lung cancer (NSCLC), which accounts for approximately 85% of lung cancers, and small cell lung cancer (SCLC). Lung adenocarcinoma and lung squamous cell carcinoma are the main subtypes of NSCLC. The common clinical therapeutic approaches for NSCLC are surgery, chemotherapy, radiotherapy and other methods. With the development of molecular targeted therapy and biological immunotherapy, the 3‐year survival rate of NSCLC has increased to 38%, but the mortality rate remains high relative to that of other cancers. Moreover, the overall cancer mortality rate is still largely driven by lung cancer.^1^ Metastasis, the main cause of death in NSCLC patients,^2^ is closely related to glycolysis.^3^

The proliferation and migration of tumour cells require a large energy supply, and glucose metabolism is the main source of energy production in humans. Glycolysis is considered a major pathway of glucose metabolism in cancer cells. Glycolysis can not only generate ATP rapidly^4^ but also provide anabolic substrates to support cell proliferation and migration.^5^ In addition, activation of glycolysis leads to an acidic tumour microenvironment and affects tumour metastasis. To adapt to an acidic microenvironment, tumour cells need to acquire an acid‐induced cytotoxicity phenotype and acquire the capacity for glycolysis and acid resistance, and these changes confer a strong growth advantage by generating the characteristics of uncontrolled metastasis.^6^, ^7^, ^8^ The highly glycolytic phenotype of NSCLC is positively correlated with the degree of malignancy and the characteristics of metastasis. Targeting tumour metabolism has thus become a promising approach for cancer treatment.^9^

NSCLC metabolism is highly heterogeneous,^10^, ^11^ and metabolic therapy has gained increasing interest in regards to NSCLC.^12^ Research showed that the differentially expressed metabolism‐related genes between the metastatic and nonmetastatic states of lung adenocarcinoma were the genes most strongly associated with metastasis‐specific lethality.^13^, ^14^ The metastasis of lung adenocarcinoma was closely related to metabolic characteristics. The metabolic heterogeneity of NSCLC affects the response to first‐line drug treatments and prognosis. For example, cisplatin‐resistant lung cancer cells have metabolism‐driven metastasis accompanied by a decreased treatment response.^15^ Metabolic heterogeneity not only affects NSCLC occurrence and development but also is a potential target for NSCLC treatment. The recognition of the specific metabolic phenotype and inherent metabolic characteristics of NSCLC patients have led to a breakthrough in the development of precise targeted metabolic therapies and combination therapies.^15^

Antitumour drugs targeting glycolysis, such as 2‐deoxyglucose (2‐DG), 3‐bromopyruvate (3‐BP) and 2‐chloroacetic acid, are used in clinical studies. Due to the lack of well‐defined molecular targets, the preclinical effect of these drugs was significant, but the clinical effect was unsatisfactory.^16^ With further research on the impact of metabolism on NSCLC progression, it is possible to search for new drugs that limit metabolism during tumorigenesis, progression and metastasis.^17^, ^18^, ^19^, ^20^

Pirfenidone (PFD) has been approved for the clinical treatment of idiopathic pulmonary fibrosis (IPF) first in the United States and then in Europe. PFD exerts anti‐fibrotic effects by significantly inhibiting TGF‐β1.^21^, ^22^ The enhanced glycolytic activity in IPF patients is similar to that in patients with NSCLC.^21^, ^22^ TGF‐β1 was found to be abnormally activated in human NSCLC tissues,^23^ and its role in the tumour microenvironment is complex and paradoxical. TGF‐β1 can regulate the activity of key glycolytic enzymes.^24^ Recent studies have found that PFD exhibits antitumour activity in addition to its known anti‐fibrotic functions. In A549 cells stimulated with TGF‐β1, PFD reversed changes in epithelial gene expression.^25^ In NSCLC, PFD exerted synergistic effects on cell death by promoting apoptosis in tumour cells and tumour‐associated fibroblasts^26^ and inhibiting tumour cell motility.^27^ In lung cancer patients with preexisting IPF, PFD altered the tumour immune microenvironment to increase the therapeutic effect of programmed death ligand 1 (PD‐L1) blockade.^28^ PFD exerts an antitumour effect by inhibiting TGF‐β1,^22^, ^29^, ^30^, ^31^ but its effect on metabolism in NSCLC and the associated mechanism are still unclear.

The main therapeutic target of PFD is TGF‐β1, which is the classical EMT promoter. Studies have found that EMT is closely related to tumour metabolism.^32^, ^33^ Our experiments showed that PFD inhibited glycolysis in NSCLC cells. We thus wondered whether PFD could inhibit glycolysis by targeting TGF‐β1—and if so, what was the underlying mechanism in NSCLC. Here, we explored the effect of PFD on different metabolic phenotypes of NSCLC cells and investigated its glycolysis‐associated mechanism in vitro and in vivo through studies in five human NSCLC cell lines and animal models and attempted to use PFD to chemosensitize NSCLC by targeting metabolism. We further investigated whether PFD could increase the therapeutic sensitivity to clinical chemotherapy drugs by modulating glycolysis in specific types of NSCLC to provide a reference for precise clinical targeted metabolic therapy programs.

## 2 MATERIALS AND METHODS

### 2.1 Cell culture

All cell lines were incubated with 10% foetal bovine serum (FBS, Wisent, Australia) and 1% penicillin and streptomycin (Gibco, US) at 37°C in 5% CO_2_. H1299 and A549 cells were both purchased from the Chinese Academy of Sciences (CAS, Shanghai, China), and H23, H520 and SK‐MES‐1 cells were purchased from the American Type Culture Collection (ATCC). A549 cells were cultured in high‐glucose DMEM (Gibco, US); H1299, H23 and H520 cells were cultured in RPMI 1640 medium (Gibco, US); and SK‐MES‐1 cells were cultured in MEM (Gibco, US). Cells in the exponential growth phase were used for experiments.

### 2.2 CCK8 assay

Cells were seeded at a density of 10^3^ cells/well in 96‐well plates in triplicate. 10 μL of CCK8 solution (Dojindo Laboratories, Kumamoto, Japan) was diluted to 100 μL and added to each well of the plate, which was then incubated for 1.5 h at 37 °C until the suitable density was reached and the pharmaceutical intervention was performed. The optical density was measured at 450 nm with a microplate reader to determine the cell proliferation capacity.

### 2.3 Western blotting

Cells were seeded into dishes and incubated for the indicated times, and the indicated reagents were then immediately applied for the designated times. RIPA buffer containing a complete protease inhibitor was used to prepare whole cell lysates on ice for 30 min. Protein was quantified using the Bradford method. After centrifugation at 12000 rpm and 4°C for 15 min and removal of insoluble material, equal amounts of protein were separated by electrophoresis on 10% SDS‐polyacrylamide gels at 80 V for 1 h and 120 V for 1.5 h. Proteins in the gels were transferred to PVDF membranes at 350 mA for 2 h. Membranes were blocked in 3% nonfat milk in PBST (PBS containing 0.1% Tween 20), washed three times in 0.1% TBST for 20 min and incubated with the indicated primary antibodies overnight at 4°C following the manufacturer's instructions. Membranes were then incubated with HRP‐conjugated secondary antibodies at room temperature for 2 h. Signals were detected with enhanced chemiluminescence reagents by standard western blot protocols.

### 2.4 Immunofluorescence staining

Cells were treated in accordance with the experimental design. The cells were washed in precooled PBS and fixed with 4% paraformaldehyde for 15 min. A series of three washes in PBS for 5 min each was followed by 15 min of permeabilization using 0.1% Triton X‐100. The cells were blocked in PBS containing 5% BSA for 55 min and incubated with primary antibodies overnight at 4°C. The cells were then washed with PBS three times for 5 min each and incubated with the secondary antibody (diluted 1:50) in PBS for 1 h at 37°C. The cells were washed again four times for 5 min each with PBS and were then incubated with DAPI for 10 min, treated with an anti‐fluorescence quenching agent and observed by fluorescence microscopy.

### 2.5 Glucose and lactate assays

Glucose and lactate concentrations were measured in fresh culture medium after drug intervention using the Glucose Test Kit (Nanjing Jiancheng Bioengineering Institute, Nanjing, China) and the Lactate Assay Kit (*Nanjing* Jiancheng Bioengineering Institute), respectively, according to the manufacturer's protocol, after the cells were harvested and counted. The concentrations of glucose and lactate were calculated as the concentration per 100 cells. All values were normalized based on the cell number.

### 2.6 Scratch assay

After cells were seeded into 6‐well plates and grown to confluence, a sterile pipette tip was used to create an even linear scratch in each well. The cells were treated as indicated in the designed experiments, and the medium was replaced with serum‐free medium. The wound coverage areas were observed and photographed under a microscope after the cells were cultured at 37°C in a CO_2_ incubator containing 5% CO_2_ for the predetermined times. The leading edge of cell coverage was measured and quantified.

### 2.7 Colony formation assay

A colony formation assay was performed to evaluate the clonogenicity of single cells. Cells were cultured in 6‐well plates at a density of 1000 cells/well, and drug intervention was performed after the cells had adhered to the plate wall. The medium was replaced after drug intervention. After 2 weeks, proliferating colonies were visible to the naked eye, and these colonies were washed twice with PBS and fixed for 10 min with 4% paraformaldehyde.

### 2.8 Apoptosis and cell cycle assay

The cells cultured in 6‐well dishes were collected with 0.25% trypsin and washed with precooled PBS. The experiment was carried out using the cell cycle ([Keygen, Nanjing, China] and apoptosis [Dojindo, Japan]) analysis kit. Cells needed attachment and fixation with 70% ethanol overnight just in cell cycle detecting. After centrifugation (1500 rpm, 5 min), the cells were resuspended in binding buffer, and staining was performed according to the kit instructions. FACS was used quantify the cell cycle distribution (the percentages of cells in G0, G1, S, and G2/M phases) and apoptosis by flow cytometry.

### 2.9 Animals and xenograft assay

Animals: BALB/c nude mice (male, 4 weeks, 18–20 g) were purchased from the Experimental Animal Center of Guizhou Medical University (Guizhou, China). Animal experiments were ethically approved by the Animal Care Welfare Committee of Guizhou Medical University (registration no. 2200494).

Xenograft tumour formation assay: Fifty male BALB/c nude mice were housed under specific pathogen‐free (SPF) laboratory conditions with adaptive feeding for 1 week and free access to food and water. The mice were randomly divided into 10 groups (*n* = 5 mice per group). Mice in eight of these groups were injected with 4 × 10^6^ A549 (4 groups) or H1299 (4 groups) cells subcutaneously in the region of the right axilla. Mice in the remaining two groups were injected with 4 × 10^6^ A549 (1 group) or H1299 (1 group) cells both pretreated with PFD (2 mmoL/L, 24 h). After 2 weeks of subcutaneous tumorigenesis, experiments were performed as designed (intraperitoneal administration of 200 mg/kg PFD once per day and cisplatin twice per week) for 4 weeks. Mouse weights and tumour sizes were recorded using a scale and a calliper, respectively, twice per week. Tumour volume was calculated as 0.5 × L × W^2^ (W represents the width; L represents the perpendicular width at the widest point). After the mice were sacrificed, tumours were removed and were weighed. Half of the tumours were fixed using 4% paraformaldehyde, and the other half were stored at −80°C for subsequent experiments.

### 2.10 Immunohistochemical (IHC) staining

Paraffin‐embedded tissue sections were deparaffinized with xylene for 30 min and rehydrated through a graded ethanol series (100%, 90%, 80%, 70% and 50%) and H_2_O for 5 min each. The sections were placed in sodium citrate buffer for antigen retrieval for 20 min (with microwave heating). Binding of nonspecific antigens was blocked in strict accordance with the kit instructions. The sections were incubated with a primary antibody overnight at 4°C in a humidified chamber. The sections were then incubated in a 37°C water bath for 30 min and washed again three times for 3 min each with PBS. Then, secondary antibody incubation was conducted at 37°C for 30 min. Next, DAB staining and haematoxylin counterstaining were performed in strict accordance with established protocols. Finally, after dehydration through an ethanol gradient (70%, 85%, 95%, 100% and 100%), the slides were sealed and photographed.

### 2.11 Energy metabolomics analysis by HPLC–MS/MS

Tumour tissue samples stored in liquid nitrogen were thawed at 4°C, and then, 100 mg of each sample was added to 1 mL of cold methanol:acetonitrile:H_2_O (2:2:1). The mixture was homogenized, the tissue was lysed and ultrasonicated twice, and the mixture was then redissolved in 100 μL of acetonitrile: water (1:1). The solution was vortexed and centrifuged (14,000 × g, 4°C, 15 min). The supernatants were collected for HPLC–MS/MS analysis on an Agilent Technologies UHPLC system (1290 Infinity LC) connected to a QTRAP LC–MS system (AB Sciex 5500). The detection parameters were as follows: sampling temperature, 4°C; column temperature, 45°C; flow rate, 300 μL/min; and loading quantity, 2 μL (one quality control (QC) run was performed after every 5 samples). Multiple reaction monitoring (MRM) mode was used for detection (negative electrospray ionization [ESI] mode determined by the analysis of metabolite standards [100 mg/mL]). Metabolite retention times were determined by the corresponding MRM (Q1/Q3) transition. The samples were measured together with a standard mixture sample (32 STD‐mix) that included all 32 tested energy metabolites. The data were acquired and processed by MultiQuant software and were then analysed by boxplot generation and hierarchical clustering.

### 2.12 Antibodies and reagents

Antibodies specific for HK2 (ab209847, Abcam), PKM2 (#3198, CST), LDHA (ab101562, Abcam), GLUT1 (ab115730, Abcam), β‐actin (#4967, CST), TGF‐β1 (ab215715, Abcam), E Cadherin (ab231303, Abcam), N Cadherin (ab98952, Abcam), Vimentin (ab8978, Abcam) and Ki67 (ab15580, Abcam) were used. Recombinant human TGF‐β1 was obtained from PeproTech (0216209, USA). A TGF‐β receptor type I/II inhibitor (MedChemExpress, LY2109761, USA) and PFD (Sigma, Germany) were used.

### 2.13 Statistical analysis

The data were input into Excel and analysed by SPSS 20.0. One‐way analysis of variance (anova) was used to determine the significance of differences among more than two groups. The LSD test was used when the variance was homogeneous, and Dunnett's *T*3 test was used when the variance was nonhomogeneous. Differences were considered statistically significant when *p* < 0.05 and highly significant when *p* < 0.01.

## 3 RESULTS

### 3.1 NSCLC presents a high degree of metabolic heterogeneity

**FIGURE 1:**
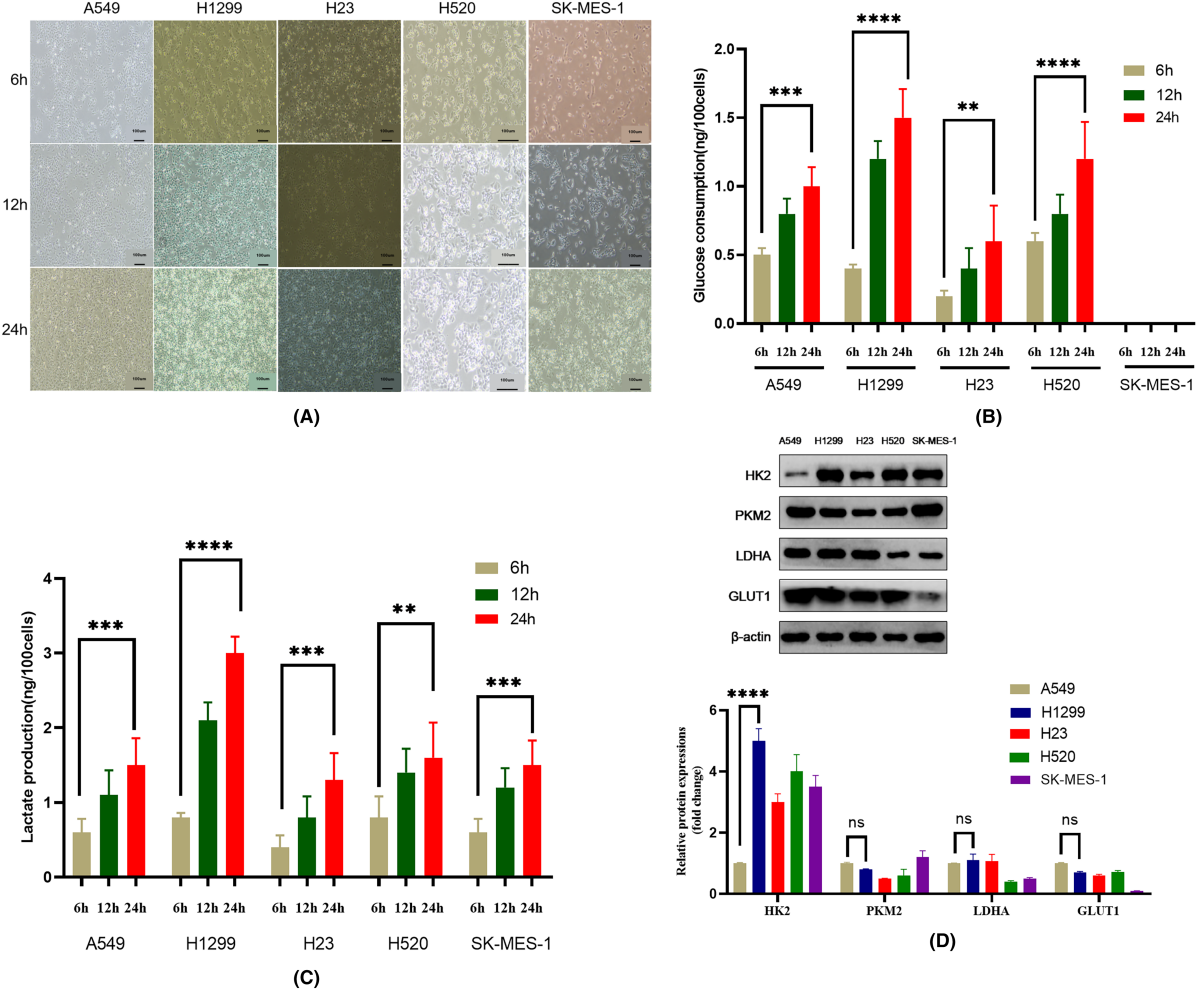
Five NSCLC cell lines presented metabolic heterogeneity. (A) Micrographs of the cells (A549, H1299, H23, H520 and SK‐MES‐1) acquired at 6 h, 12 h and 24 h in the absence of any intervention. (B, C) Glucose consumption and lactate output at 6, 12 and 24 h. (D) Analysis of the expression of glycolysis‐related enzymes in the five NSCLC cell lines by western blotting. All data are shown as the means ± SDs; *p < 0.05, **p < 0.01, ***p < 0.001, ****p < 0.0001, *n* = 3.

To completely understand the metabolic characteristics of different NSCLC cells and provide a basis for subsequent studies, we selected five human NSCLC cell lines (A549, H1299, H23, H520 and SK‐MES‐1) with different metabolic phenotypes for the experiment. Among these cell lines, A549, H1299 and H23 are lung adenocarcinoma cell lines, and H520 and SK‐MES‐1 are lung squamous cell carcinoma cell lines. These cell lines represent the pathological subtypes of lung adenocarcinoma and lung squamous cell carcinoma with different degrees of malignancy. A549 is a wild‐type lung adenocarcinoma cell line, H1299 is a p53(−) lymph node‐metastatic lung adenocarcinoma cell line, H520 is a wild‐type lung squamous cell carcinoma cell line, H23 is a K‐ras and p53 mutant lung squamous cell carcinoma cell line, and SK‐MES‐1 is a cell line derived from pleural fluid from a patient with metastatic lung squamous cell carcinoma. We plated these five NSCLC cell lines at an equal density, observed their growth and measured their glucose consumption and lactate production at 6 h, 12 h and 24 h to assess their glycolytic activity. All five NSCLC cell lines produced large amounts of lactate via glycolysis and had different glucose consumption capacities (Figure 1B,C). Their degree of glycolysis correlated with their morphology and growth rate (Figure 1A). In addition, the glucose consumption and lactate output of H1299 cells were approximately twofold greater than those of A549 cells (Figure 1B,C). H1299 and SK‐MES‐1 cells proliferated faster, demonstrating that within the same pathological subtype, metastatic cells with a higher degree of malignancy proliferate faster. Notably, both glucose uptake and lactate production in lymph node‐metastatic H1299 lung adenocarcinoma cells were higher than those in the other NSCLC cells. Glycolytic activity was highest in metastatic cells of lung adenocarcinoma, suggesting a closer relationship between glycolysis and metastasis in lung adenocarcinoma than in lung squamous cell carcinoma. Interestingly, SK‐MES‐1 cells produced lactate without glucose because the recommended medium for these cells, MEM, contains no sugars. SK‐MES‐1 cells are pleural metastatic lung squamous cell carcinoma cells, and a pleural effusion is a liquid environment containing no sugars. To determine whether SK‐MES‐1 cells are forced to consume other substances to produce lactate or cannot utilize glucose, we changed the culture medium to high‐glucose DMEM and found that the cells grew and produced large amounts of lactate without consuming glucose. Thus, the glycolytic phenotype of SK‐MES‐1 cells is lactate production without glucose consumption. This phenotype might be related to the environment of pleural effusions, which is also an environment containing no sugar in humans. SK‐MES‐1 cells might adopt a lactate metabolism bypass pathway to produce lactate, indicating that NSCLC cells have high metabolic heterogeneity and that there are dynamic synergistic mechanisms among glucose metabolism pathways in humans to meet the energy needs of the body.

Glycolytic ability is determined mainly by the activity of glycolysis‐related enzymes. We further performed western blotting to measure the protein expression of glycolysis‐related enzymes and found that the protein expression of the key glycolytic enzyme HK2 in H1299 cells was approximately fivefold that in A549 cells (Figure 1D) and significantly higher than that in the other five NSCLC cell lines in the absence of any intervention.

HK2 is the first key enzyme in glycolysis and plays an important role in regulating glycolysis, consistent with the results of the glucose consumption and lactate production assays. NSCLC has a high degree of metabolic heterogeneity, and both primary and metastatic cancers have a glycolytic metabolic phenotype, which is closely related to growth and metastasis (Figure 1A–C).

### 3.2 PFD inhibits the proliferation of NSCLC cells by targeting the key glycolytic enzymes HK2, LDHA and GLUT1 to inhibit glycolysis

**FIGURE 2:**
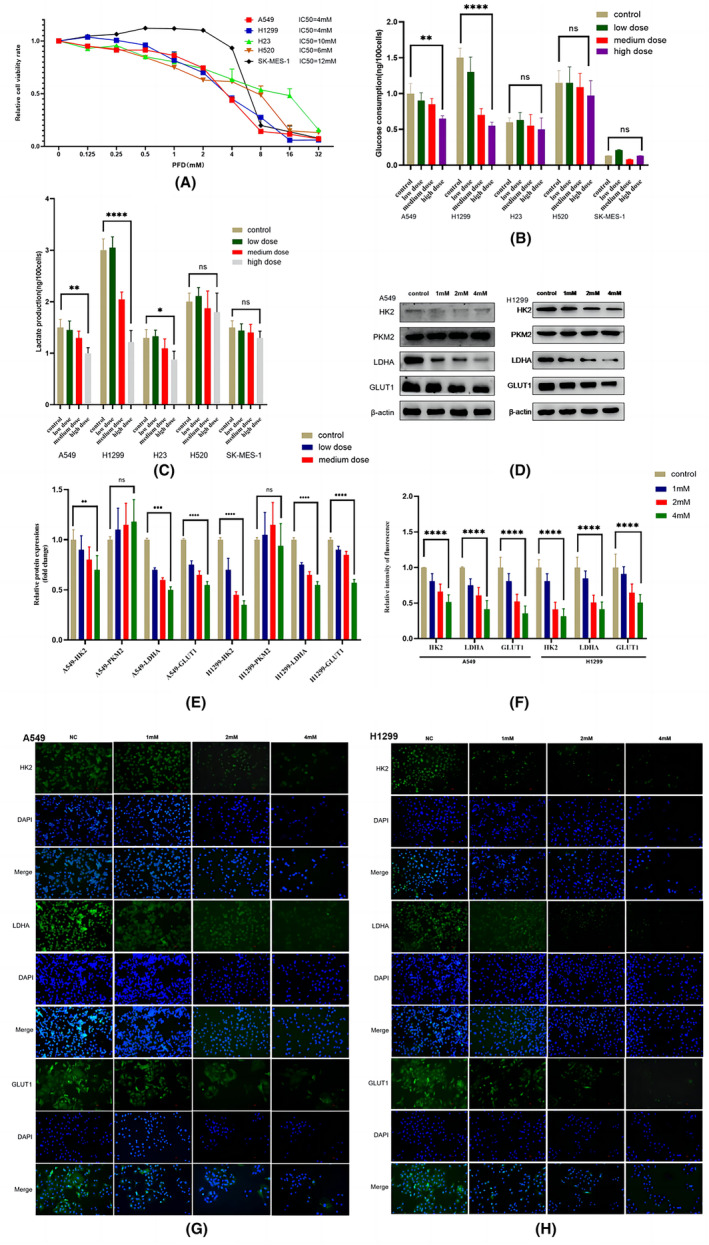
PFD inhibited cell proliferation and glycolysis. (A) Viability rate of five NSCLC cell lines (A549, H1299, H23, H520, and SK‐MES‐1) after treatment with different concentrations of PFD for 24 h; IC50 of PFD. (B, C) Glucose consumption and lactate output after treatment with low (1/4 IC50), medium (1/2 IC50) and high (IC50) concentrations of PFD for 24 h. (D, E) Expression levels of glycolysis‐related enzymes in A549 and H1299 cell lines treated with PFD at different concentrations for 24 h, as determined by western blotting. (F) Histogram of the data in (G, H). (G, H) Confirmation of western blot results by immunofluorescence staining. All data are shown as the means ± SDs; *p < 0.05; **p < 0.01; ***p < 0.001; ****p < 0.0001, *n* = 3.

A CCK8 assay was used to test the effect of PFD on the proliferation activity of five NSCLC cell lines at 10 concentrations ranging from 0 to 32 mmol/L. The experimental results showed that cell viability was significantly reduced by PFD treatment. PFD inhibited the proliferation of NSCLC cells (Figure 2A). Based on the half‐maximal inhibitory concentration (IC50) value obtained from the proliferation curve, the IC50 of PFD in H1299 cells was 4 mmol/L, which was the lowest among the five cell lines, suggesting that the metastatic lung adenocarcinoma line might be the most sensitive to PFD. To further explore the effect of PFD on glycolysis in NSCLC, we selected the 1/4 IC50, 1/2 IC50 and IC50 values and divided the cells into the low‐dose, medium‐dose and high‐dose groups, respectively, and measured the glucose and lactate contents in the final culture supernatant after 24 h of treatment. PFD treatment affected glycolysis of lung adenocarcinoma cells but not lung squamous carcinoma cells, as determined by the glucose consumption and lactate output measurements (Figure 2B,C). To further verify the causes of the glycolytic changes, we detected the changes in the expression of the main glycolysis‐related enzymes (HK2, PKM2, LDHA and GLUT1) in these five NSCLC cell lines after treatment with PFD (Figure 2D,E, Figure S1). The protein expression of glycolysis‐related enzymes (HK2, LDHA and GLUT1) was confirmed to be decreased in PFD‐treated lung adenocarcinoma cells (A549 and H1299) but not in PFD‐treated lung squamous carcinoma cells by western blotting (Figure 2D,E, Figure S1). To further verify the results of western blotting, we performed immunofluorescence staining in A549 and H1299 cells, and the results were consistent with those of western blotting (Figure 2F,G,H). The PFD‐induced changes in the protein levels of glycolysis‐related enzymes were consistent with the effect of PFD treatment on glycolysis (glucose consumption and lactate output). The above experimental results suggested that PFD inhibited glucose consumption and lactate production via glycolysis by inhibiting the activity of key glycolytic enzymes (HK2, LDHA and GLUT1) and inhibited cell proliferation. This phenomenon occurred only in lung adenocarcinoma cells, indicating that the mechanisms by which drugs inhibit the growth of NSCLC cells with different metabolic characteristics are different. Thus, the metabolic characteristics of the NSCLC tumour should be fully considered when selecting drugs for metabolic therapy.

### 3.3 PFD exerts different proapoptotic and cell cycle arrest effects to exert antitumour activity

**FIGURE 3:**
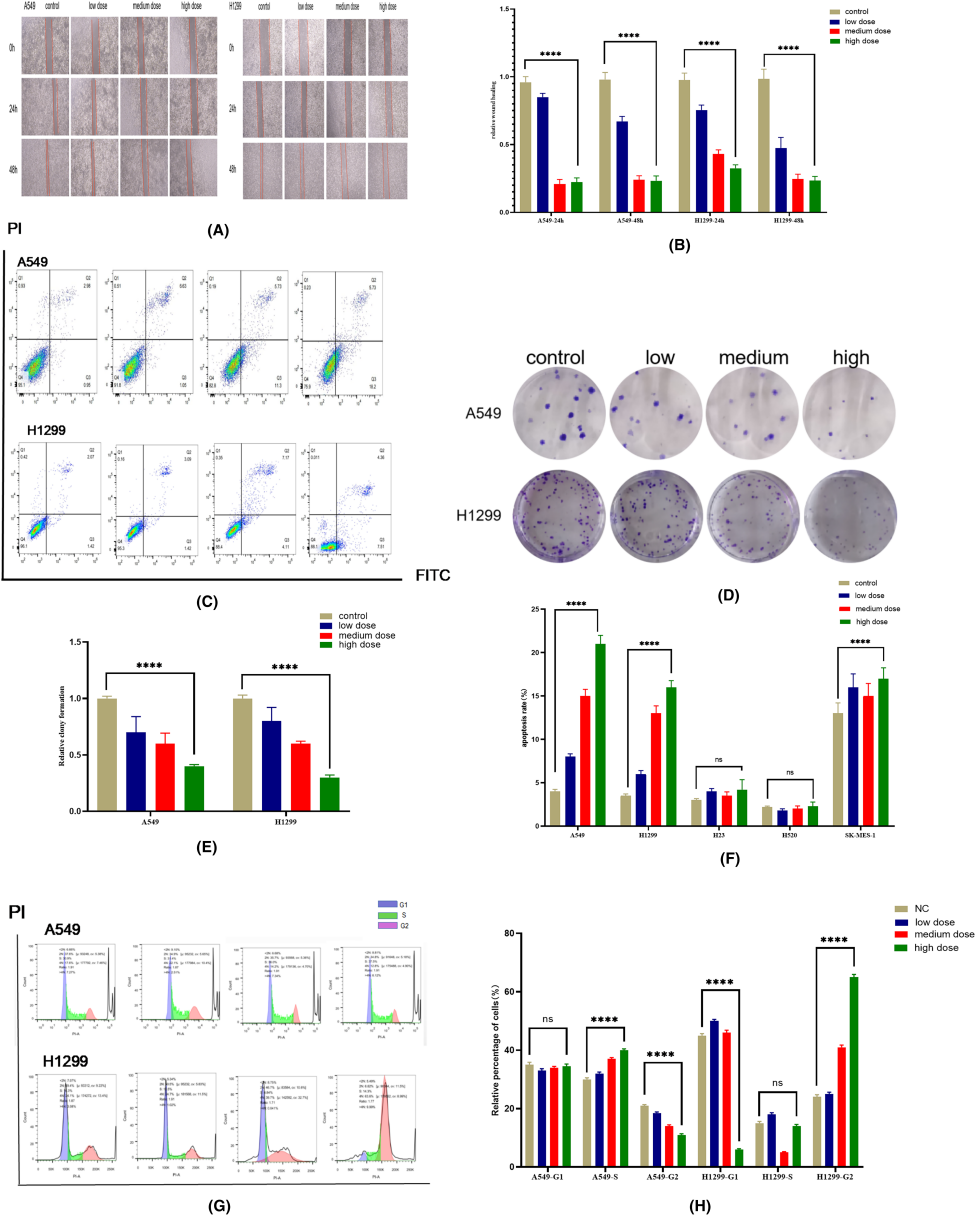
PFD had different antitumour effects on A549 and H1299 cells with different metabolic phenotypes. (A) Scratch assay of cells treated with different concentrations of PFD for 24 h and 48 h. (B) Histogram of the data in a. (C) Apoptosis after treatment with different concentrations of PFD for 24 h. (D) Colony formation assay of cells treated with different concentrations of PFD for 2 weeks. (E) Histogram of the data in d. (F) Histogram of the data in c. (G) Cell cycle distribution after treatment with different concentrations of PFD for 24 h. (H) Histogram of the data in g. All data are shown as the means ± SDs; *p < 0.05; **p < 0.01; ***p < 0.001; ****p < 0.0001, *n* = 3.

To understand how PFD influences the development of NSCLC, we carried out a series of experiments to investigate its effects on cell migration and colony formation. The results of the scratch assay showed that PFD inhibited cell migration at both 24 h and 48 h. The colony formation assay is an important method to measure cell proliferation. PFD reduced the size and number of stained single cell‐derived colonies after culture for 2 weeks. Moreover, lung adenocarcinoma A549 and H1299 cells formed large clonal communities, and PFD inhibited the migration and colony formation of these cells (Figure 3A,B,D,E), but not effected the lung squamous cell carcinoma (Figures S2 and S3).

Metastasis and tumour growth are closely related to apoptosis and cell cycle progression. Thus, we evaluated changes in apoptosis and the cell cycle after PFD treatment by flow cytometry. PFD had a proapoptotic effect on A549 and H1299 lung adenocarcinoma cells, especially on early apoptosis in H1299 cells, as shown by flow cytometry (Figure 3C,F). When studying the antitumour effects of drugs, more attention should be given to the effect on early apoptosis, as early apoptosis is more closely related to metabolic stress. Furthermore, consistent with its effect on apoptosis in lung adenocarcinoma cells, PFD induced different degrees of cell cycle arrest in A549 and H1299 lung adenocarcinoma cells; the high concentration of PFD induced obvious G2 arrest in metastatic H1299 lung adenocarcinoma cells, and the number of cells in G2 phase increased approximately threefold (Figure 3G,H). These results indicated that in addition to its inhibition of glycolysis, PFD exerted different antitumour effects on different pathological types of NSCLC and obvious effects on promoting apoptosis and inducing cell cycle arrest in lung adenocarcinoma cells. Even in the same pathological type of NSCLC, PFD has different effects on promoting apoptosis and inducing cell cycle arrest in lung adenocarcinoma cells with different metabolic characteristics.

### 3.4 PFD inhibits EMT by increasing E cadherin expression, especially in NSCLC cells with a highly metabolic phenotype

**FIGURE 4:**
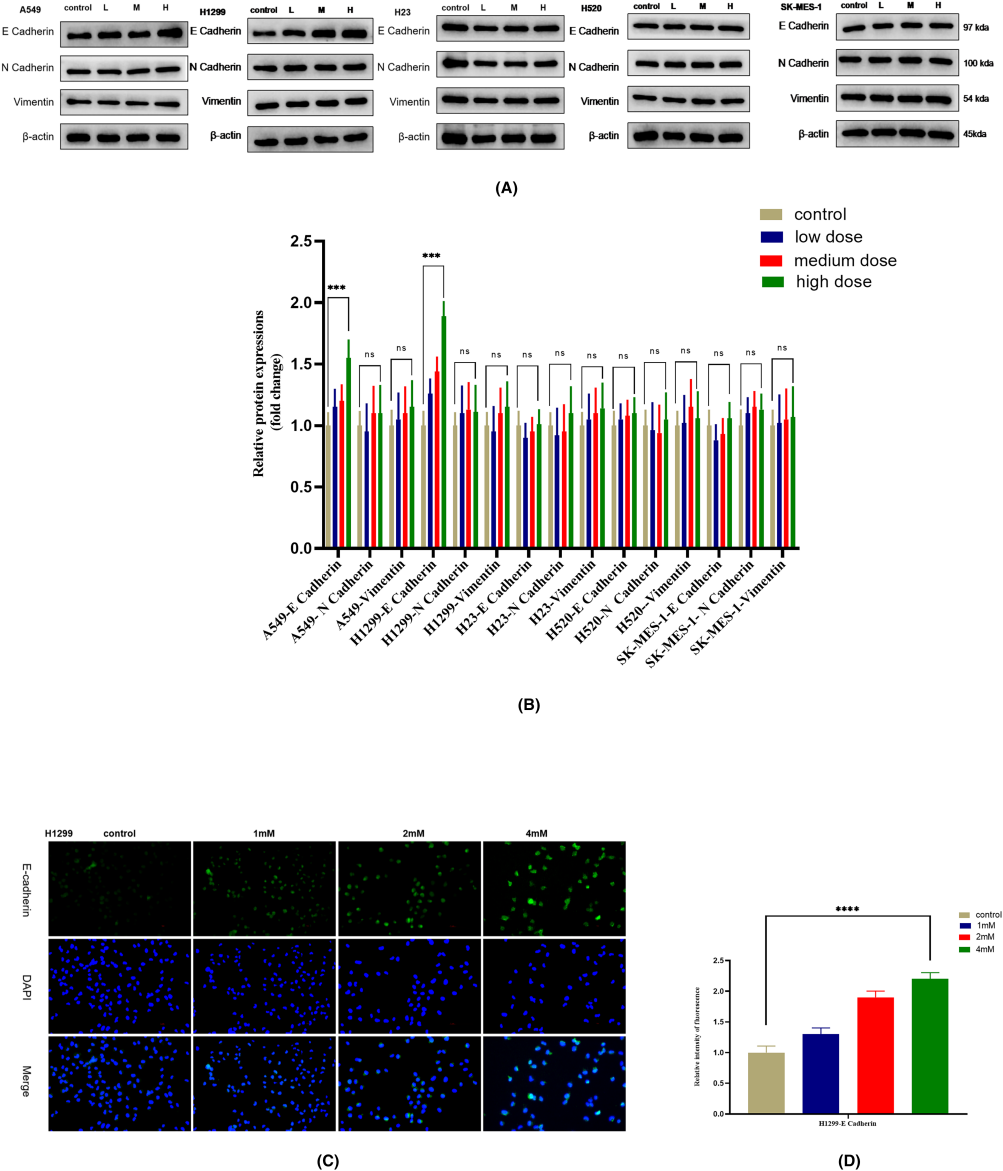
PFD increases the expression of E cadherin. (A) The expression of EMT marker proteins after PFD treatment (24 h) at different concentrations, as measured by western blotting. (B) Histogram of the data in a. (C) Confirmation of the western blot results by immunofluorescence staining. (D) Histogram of the data in c. All data are shown as the means ± SDs; *p < 0.05; **p < 0.01; ***p < 0.001; ****p < 0.0001, *n* = 3.

Through the above experiments, we found that the effect of PFD on glycolysis and its other antitumour effects were more obvious in lung adenocarcinoma cells with a highly metabolic phenotype. Glycolysis is the main pathway of tumour metabolism, and it can not only increase the survival and proliferation ability of tumour cells but also promote tumour metastasis by generating large quantities of metabolic intermediates. High glycolytic activity is closely related to the characteristics of tumour cell metastasis, and EMT is an important phenotype promoting tumour metastasis. We thus sought to determine whether PFD exerts similar effects on EMT in NSCLC cells. We detected the expression changes in the main marker proteins of EMT (E cadherin, N cadherin and Vimentin) by western blotting. Among these proteins, N cadherin and vimentin are the markers of mesenchymal cells, and E cadherin is the marker of epithelial cells. Consistent with our observation of decreased glycolysis in response to PFD treatment in NSCLC cells, PFD increased the protein expression of the epithelial marker E cadherin to inhibit EMT (Figure 4A,B), especially in lymph node‐metastatic lung adenocarcinoma H1299 cells, which have a highly glycolytic phenotype. To further verify the results of western blotting, we performed immunofluorescence staining, and the result was consistent with that of western blotting (Figure 4C,D). These results suggested that PFD might play an antitumour role in lung adenocarcinoma cells with high metabolic activity through a synergistic effect on EMT and glycolysis, but the target and mechanism were still unclear.

### 3.5 PFD inhibits glycolysis and EMT by targeting TGF‐β1 to play an antitumour role, especially in NSCLC with a highly metabolic phenotype

**FIGURE 5:**
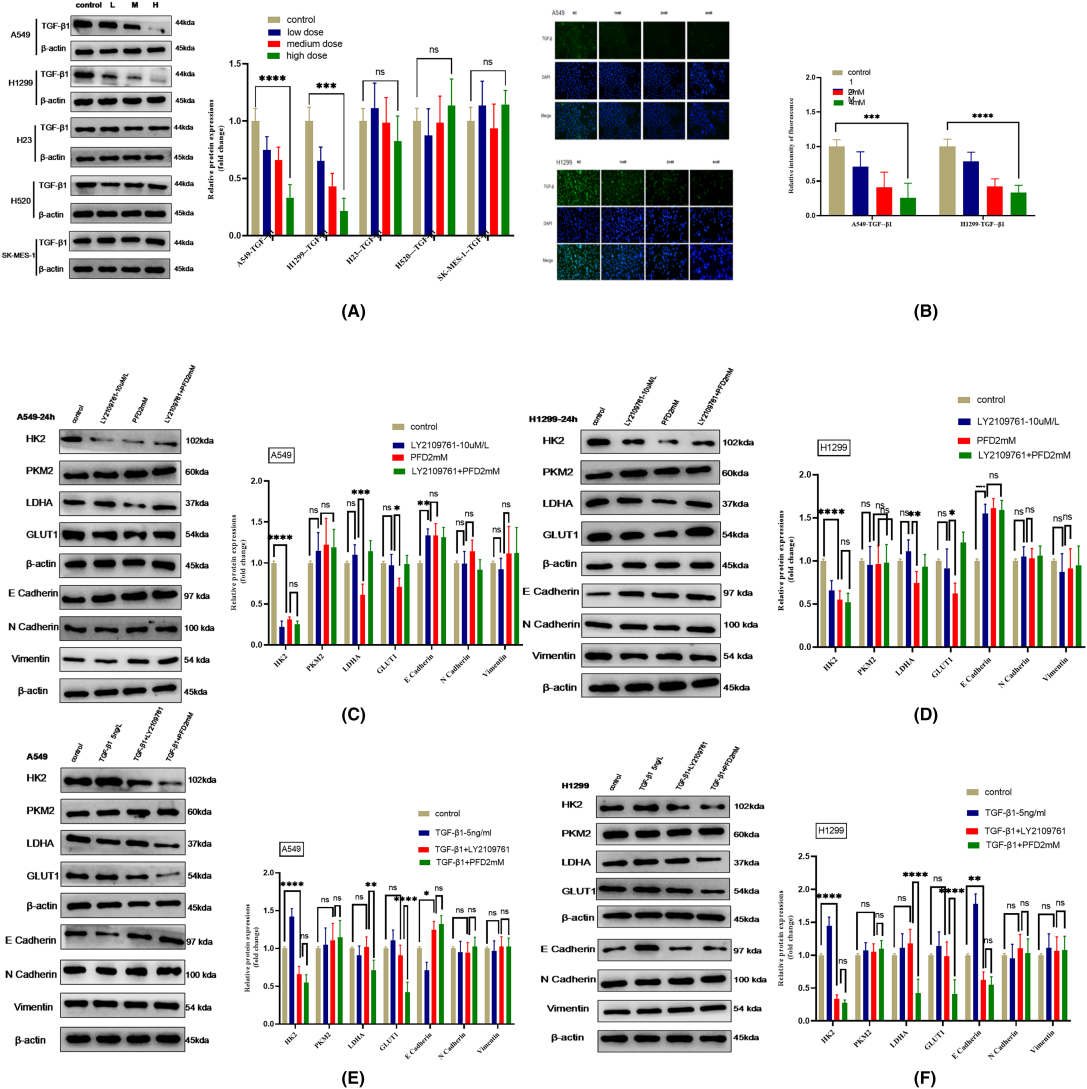
PFD inhibits the crosstalk between glycolysis and EMT by targeting TGF‐β1. (A) TGF‐β1 expression after PFD treatment (24 h) at different concentrations, as determined by western blotting. (B) Confirmation of the western blot results in a by immunofluorescence staining. (C, D) The protein expression of glycolytic enzymes and EMT markers after TGF‐β receptor inhibitor treatment in A549 and H1299 cells. (E, F) The protein expression of glycolytic enzymes and EMT markers after TGF‐β1 treatment in A549 and H1299 cells. All data are shown as the means ± SDs; *p < 0.05; **p < 0.01, ***p < 0.001; ****p < 0.0001, *n* = 3.

PFD plays an evident anti‐inflammatory and anti‐fibrotic role by targeting TGF‐β1. Inflammatory cells and fibrotic cells have metabolic characteristics similar to those of tumour cells. Therefore, we sought to determine whether PFD inhibits glycolysis and EMT by targeting TGF‐β1. We found by both western blotting and immunofluorescence staining that the protein expression of TGF‐β1 in lung adenocarcinoma cells decreased progressively in a dose‐dependent manner with increasing PFD treatment time in culture (Figure 5A,B), suggesting that PFD might play an antitumour role by targeting TGF‐β1.

To verify whether PFD could inhibit glycolysis and EMT when TGF‐β1 activity was inhibited, we evaluated the effects of PFD after TGF‐β receptor inhibitor (LY2109761) pretreatment. The efficacy of TGF‐β1 suppression after treatment with LY2109761 was verified, and we selected the optimal concentration and time (10 μM and 24 h) through assessment of proliferation curves generated during CCK8 assays (Figure S3), and used these parameters in follow‐up experiments to inhibit TGF‐β1 function. We used LY2109761 and PFD alone and in combination to compare their effects on glycolysis and EMT. Similar inhibition of glycolysis and EMT by PFD and LY2109761 treatment was observed, as evidenced by CCK8 assay and western blot results (Figure 5C,D). Consistent with the findings after PFD treatment, the protein expression of the critical glycolytic enzyme HK2 was decreased, and the expression of an EMT marker protein (E cadherin) was increased by LY2109761 treatment, as shown by western blotting; however, LY2109761 did not affect LDHA or GLUT1 expression (Figure 5C,D). The combined effect of LY2109761 and PFD was not obviously better than that of either drug alone. Due to the limited number of TGF‐β1 receptors in cells, the combined effect of PFD and LY2109761 was not significantly better than the effect of either drug alone, and PFD might inhibit glycolysis and EMT by targeting TGF‐β1.

To determine whether TGF‐β1 can promote cell proliferation, we treated cells with TGF‐β1 at different concentration and for different times and evaluated cell proliferation by a CCK8 assay and expression of glycolytic‐related enzymes and generated by WB. TGF‐β1 treatment at 5 ng/mL for 24 h promoted cell proliferation most significantly (Figure S4). We then selected the optimal dose and time (5 ng/mL and 24 h) for further research to simulate the tumour microenvironment in vivo. In contrast to the findings after PFD treatment, the western blot results showed that the protein expression of the critical glycolytic enzyme HK2 was markedly increased and the protein expression of the EMT marker E cadherin was decreased by TGF‐β1 treatment; however, the expression of neither LDHA nor GLUT1 was affected (Figure 5E,F). To repeatedly verify the target and mechanism of PFD, we readjusted the drug groups. The effects of treatment with a TGF‐β receptor inhibitor (LY2109761) and PFD after TGF‐β1 pretreatment were compared. Both the TGF‐β receptor inhibitor and PFD decreased HK2 and increased E cadherin expression, but LDHA and GLUT1 expression was decreased only by PFD (Figure 5E,F). The inhibitory effects of PFD on glycolysis and EMT were stronger and more extensive and were enhanced by TGF‐β1 pretreatment. This finding supported the idea that TGF‐β1 is the critical target of PFD and that PFD plays an antitumour role by targeting TGF‐β1 to inhibit glycolysis and EMT in lung adenocarcinoma, especially in NSCLC with a highly metabolic phenotype.

### 3.6 PFD synergizes with cisplatin to exert an antitumour effect in vivo

**FIGURE 6:**
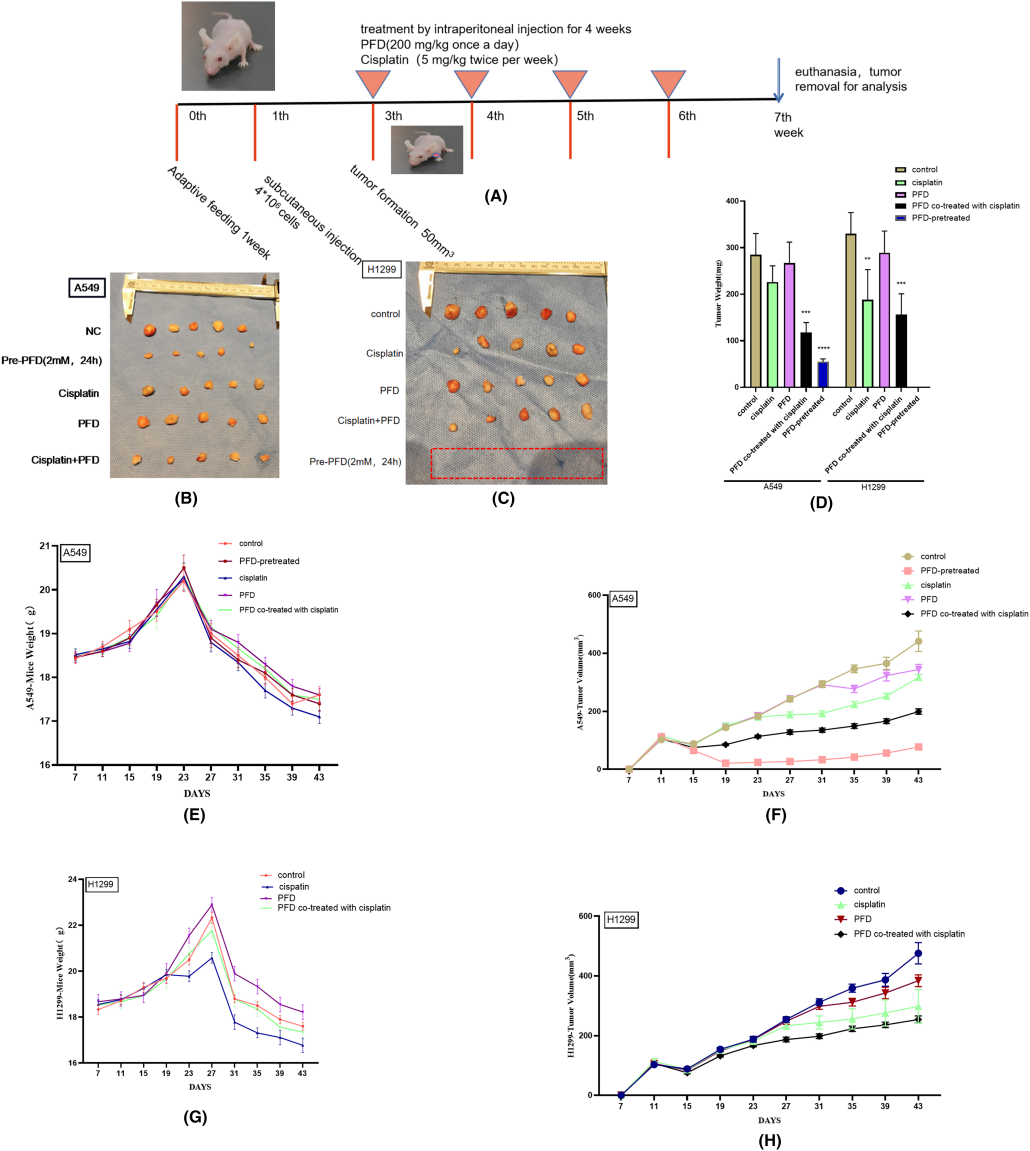
PFD synergizes with cisplatin to exert antitumour effects in vivo. (A) Animal study design and drug regimen. (B, C) Photographs of the harvested subcutaneous tumours after sacrifice. (D) Histogram of tumour weights after sacrifice. (E–H) Mouse weights and tumour volumes throughout the experiment. All data are shown as the means ± SDs; *p < 0.05; **p < 0.01; ***p < 0.001; ****p < 0.0001, *n* = 5.

Cisplatin is the first‐line chemotherapy drug for NSCLC. The major clinical problem in the use of cisplatin is the inevitable decrease in drug sensitivity, and changes in metabolism have been found in cisplatin‐resistant cells. A series of in vitro experiments indicated that PFD targeting of TGF‐β1 inhibited the crosstalk between glycolysis and EMT to play an antitumour role. We considered whether PFD can synergize with cisplatin in vivo by targeting tumour metabolism to increase therapeutic sensitivity and improve the poor prognosis of patients with metastatic tumours. To further verify the synergistic antitumour effect of PFD and cisplatin in vivo, we established a subcutaneous tumorigenesis model in nude mice and designed different subgroups. We selected the cell lines with obvious effects in vitro, that is A549 and H1299, for these experiments in vivo. PFD‐pretreated cells were used for subcutaneous tumorigenesis in nude mice. The tumour volume in the model established with A549 PFD‐pretreated cells was significantly smaller than that in the normal cell model, and none of the mice implanted with H1299 PFD‐pretreated cells exhibited subcutaneous tumour formation (Figures 6B and 5C). This result suggested that the tumorigenic ability of lung adenocarcinoma cells in vivo was reduced by PFD pretreatment, especially in lymph node‐metastatic lung adenocarcinoma. The experimental intervention was carried out based on the drug regimen (Figure 6A). PFD (200 mg/kg once every other day) and cisplatin (5 mg/kg twice per week) were administered by intraperitoneal injection for 4 weeks, and the mice were sacrificed 3 days after the last treatment with PFD and cisplatin. The drug dosage used in the in vivo experiments was determined based on the reference source.^34^ Mice in the remaining groups were injected with 4 × 10^6^ A549 or H1299 cells pretreated with PFD without any other drug intervention. Compared with those in the control group, the tumour size and weight in the group cotreated with PFD and cisplatin were substantially decreased (Figures 6E–G and 5H), and there was a slight improvement in cachexia (weight loss) due to cotreatment. These findings suggested that the combination of PFD and cisplatin might increase chemosensitivity by targeting metabolism and ameliorating adverse reactions during treatment.

### 3.7 PFD in combination with cisplatin inhibits glycolysis and EMT in vivo

**FIGURE 7:**
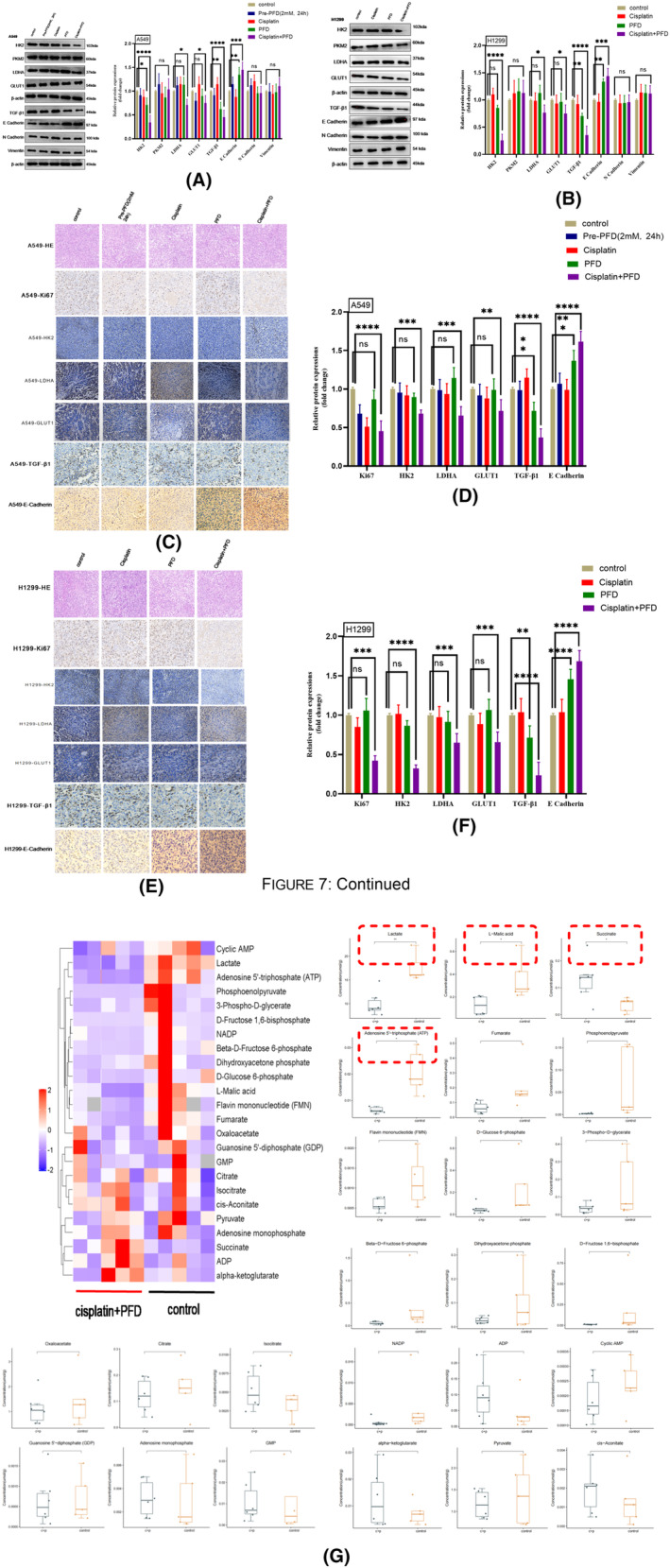
PFD synergizes with cisplatin to inhibit the crosstalk between glycolysis and EMT in vivo. (A, B) Analysis of the expression of TGF‐β1, glycolysis‐related enzymes and the classical marker of EMT by western blotting. (C, E) HE staining, immunohistochemical staining for Ki67 and confirmation of the western blot results in A and B. (D, F) Histogram of the data in c and e. (g) Targeted metabolomics analysis of glycolytic metabolites in H1299 tumour tissues. All data are shown as the means ± SDs; *p < 0.05; **p < 0.01; ***p < 0.001; ****p < 0.0001, *n* = 3.

After finding that the combination of PFD and cisplatin could inhibit tumour growth in vivo, we further clarified whether the targets and mechanisms in vivo were consistent with those in vitro. As shown by haematoxylin and eosin (HE) staining, PFD had no significant injury on tumour cell morphology or the environment of inflammatory cells compared to the control treatment. In addition, compared with that in the control group, proliferative activity was inhibited in the combination treatment group, as shown by immunohistochemical staining for Ki67 (Figures 7C–E and 5F). The combination of PFD and cisplatin exerted an antitumour effect by inhibiting cell proliferation. The protein expression levels of glycolysis‐related enzymes (HK2, LDHA and GLUT1) were reduced (Figures 7A–C and 5E), and an increase in the protein level of the classical marker of EMT, E cadherin (Figure 7A–C and 5E) also occurred, as shown by western blotting and immunofluorescence staining. Combined treatment with PFD and cisplatin inhibited glycolysis and EMT in vivo. Interestingly, the protein expression of glycolysis‐related enzymes (HK2, PKM2, LDHA and GLUT1) in the PFD‐pretreated group was not decreased compared with that in the control group, as determined by western blotting (Figure 7A), probably because PFD alters the expression of key glycolytic enzymes in a time‐dependent manner. Consistent with the inhibition of glycolysis and EMT, TGF‐β1 expression was decreased in the PFD and cisplatin cotreatment group (Figure 7A–C and 5E), as shown by immunofluorescence staining and western blotting. The above results suggested that the combination of cisplatin and PFD inhibited glycolysis and EMT by targeting TGF‐β1 and significantly improved the metabolic phenotype and cisplatin sensitivity in lymph node‐metastatic lung adenocarcinoma cells.

To fully explore the changes in metabolites in the tumour microenvironment after combination treatment with PFD and cisplatin, we conducted targeted metabolomics analysis on tissues from tumours derived from lymph node‐metastatic lung adenocarcinoma H1299 cells, in which PFD exhibited the greatest therapeutic effect. We detected 32 substances, 24 of which were confirmed. The targeted metabolomics data showed that cotreatment with cisplatin and PFD inhibited the production of the main glycolytic products, that is lactate and adenosine 5′‐triphosphate (ATP), specifically by altering the acidic tumour environment and reprogramming metabolism (Figure 7G). Consistent with the results in vitro, PFD inhibited tumour glycolysis in vivo. Also consistent with the in vitro studies, cotreatment with cisplatin and PFD inhibited tumour glycolysis and EMT in vivo by targeting TGF‐β1 and increased therapeutic sensitivity.

## 4 DISCUSSION

Due to its extremely high morbidity and mortality, NSCLC has become a major public health problem worldwide that has imposed a heavy disease burden on humans. Metastasis is a major obstacle to long‐term survival in patients with NSCLC because the disease is most often diagnosed in the middle or late stage.^2^, ^38^ Metastasis is a process in which primary cancer cells spread to secondary sites, and metabolic restriction is recognized as a suppressor of the metastatic potential of cancer cells.^8^ Metabolic remodelling is a hallmark of malignancy, and malignant cells preferentially metabolize glucose through glycolysis. Glycolysis can rapidly deprive cells of glucose and produce large amounts of lactate to generate an acidic microenvironment suitable for rapid growth and metastasis.^39^ Tumour metastasis is also closely related to metabolic reprogramming.^18^ Glycolysis can be inhibited mainly through modulation of key glycolytic enzymes to regulate tumour growth and metastasis. The acidic tumour microenvironment generated via glycolysis can influence the EMT process in cancer cells, and the EMT process and glycolytic metabolism can be coordinated to increase tumour invasion and metastasis.^9^, ^39^ Recent research found that lactate, the main product of glycolysis, was not only a simple metabolite generating the acidic microenvironment in NSCLC but also a substrate for other metabolic reactions and a molecular signal to regulate other metabolic pathways.^40^ Glycolysis plays an important regulatory role in metabolic reprogramming and determines cell fate and function.^41^ Glycolysis is associated with the degree of malignancy of tumour cells. The glycolytic efficiency of the highly metastatic breast cancer cell line MDAMB‐231 was found to be higher than that of the nonmetastatic cell line MCF‐7.^42^

In different individuals, as well as in different regions in the same individual, NSCLC appears to exhibit metabolic heterogeneity.^43^ Glycolysis, the main mode of tumour metabolism, is typically characterized by the consumption of glucose and the production of large amounts of lactate. By measuring glucose and lactate concentrations, we found that PFD inhibited glycolysis and showed different effects in cells with different metabolic characteristics. Glycolysis is not only heterogeneous but also plastic.^44^, ^45^ The effect of metabolic therapy is quite different in NSCLC, and the selection of a metabolic therapy should fully consider the metabolic characteristics of the tumour.^46^

Our experimental results suggested that PFD inhibited glycolysis and tumour growth only in lung adenocarcinoma by affecting the activity of glycolytic enzymes. Changes in metabolic enzyme activity play a crucial role not only in glycolysis but also in tumour cell proliferation, cell cycle progression and apoptosis. Apoptosis evasion is the hallmark of human cancers and promotes tumour formation and progression as well as metastasis. The proapoptotic effect was found to be beneficial in tumour.^47^, ^48^ Metabolism is closely related to cell cycle arrest in cancer cells, in addition to its canonical function of controlling cell cycle progression.^49^ Consistent with previous similar research,^50^ our data indicated that PFD played different antitumour roles, including modulating cell cycle progression, cell proliferation and apoptosis, in lung adenocarcinoma cells with different metabolic phenotypes, especially in cells with a highly glycolytic phenotype. The metabolic state of NSCLC is crucial to treatment efficacy, and metabolic therapy has an obvious effect, especially on NSCLC metabolism‐dependent cancer cells.^46^ Therefore, identifying strategies to use the metabolic characteristics of NSCLC to select a treatment plan for precisely targeted metabolic therapy to increase chemosensitivity has become a research hotspot.

The anti‐inflammatory and anti‐fibrotic effects of PFD have been extensively documented,^51^, ^52^ and PFD has been shown to exert an antitumour effect.^53^ However, there are few studies about the target of PFD and its mechanistic interface with metabolism in NSCLC.^27^, ^50^ TGF‐β1, the main target of PFD, exerts anti‐inflammatory and anti‐fibrotic effects and plays an important role in regulating key glycolytic enzymes.^54^, ^55^ TGF‐β1 participates in the crosstalk between EMT and metabolic reprogramming in cancer progression.^32^, ^56^, ^57^, ^58^ The TGF‐β1 signalling pathway is the primary driver of EMT. Moreover, the PI3K/AKT and KRAS/MEK/ERK pathways can also be triggered as effectors of noncanonical TGF‐β1 signalling, and these pathways are closely related to metabolism. A set of 44 metabolic genes collectively called the mesenchymal metabolic signature (MMS) were found to exhibit increased expression levels in high‐grade cancer cell lines, and the expression of these MMS genes was closely related to that of mesenchymal markers.^59^ Metabolism and EMT are intertwined, and TGF‐β1 may contribute to the metabolic effects associated with EMT. To investigate whether PFD could play a therapeutic role by inhibiting TGF‐β‐induced metabolic changes during EMT, we used PFD, TGF‐β1 and a TGF‐β receptor inhibitor (LY2109761) to modulate the protein expression of TGF‐β1 in multiple groups in vitro and in vivo. The effects of the TGF‐β receptor inhibitor on glycolysis and EMT were similar to those of PFD and opposite those of TGF‐β1, based on the general patterns observed. To verify the regulation of TGF‐β1 signalling by PFD, we treated lung adenocarcinoma cells with the TGF‐β receptor inhibitor and TGF‐β1 combined with PFD. PFD affected the expression of glycolysis‐related enzymes, EMT marker proteins and TGF‐β1. Costimulation with TGF‐β1 increased the efficacy of PFD, and decreasing the expression of TGF‐β1 with the TGF‐β receptor inhibitor reduced the efficacy of PFD. The effect of PFD treatment was consistent with and more extensive than that of TGF‐β receptor inhibitor treatment. This finding confirmed the hypothesis that PFD targets TGF‐β1 to inhibit glycolysis during EMT.

Cisplatin is a first‐line chemotherapy drug for the clinical treatment of NSCLC. An interesting clinical phenomenon is that metabolic alterations are associated with a decrease in drug sensitivity in metastases. Metabolism in cisplatin‐resistant NSCLC cells was reprogrammed, as evidenced by the higher metabolic activity and generation of stress.^60^ Currently, an increasing number of researchers are considering whether combinations of chemotherapy and metabolic therapy can reduce side effects and have chemosensitivity‐enhancing effects on metastatic NSCLC.^61^, ^62^ We found that PFD decreased the ability of subcutaneous tumorigenesis in nude mice. Moreover, the antitumour activity of cisplatin was enhanced by PFD cotreatment in vivo. Cotreatment with cisplatin and PFD altered metabolism by inhibiting the crosstalk between glycolysis and EMT to enhance the antitumour effect compared with that in the control and single‐treatment groups, especially in the model established with H1299 lung adenocarcinoma cells with a highly glycolytic phenotype. H1299, a lymph node‐metastatic lung adenocarcinoma cell line, is characterized by high glycolytic activity compared with that in A549 cells, a common lung adenocarcinoma cell line. Research has found that metastatic tumour cells colonizing distant sites have metabolic vulnerabilities, and the targeting of metabolism in metastatic cancer cells would constitute a breakthrough in metabolic therapy.^63^, ^64^

**FIGURE 8:**
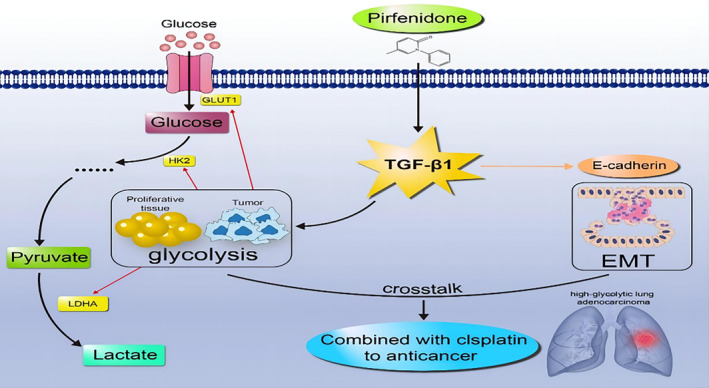
Molecular mechanism by which PFD inhibits the crosstalk between glycolysis and EMT by targeting TGF‐β1.^35^, ^36^, ^37^

In summary, PFD inhibits the crosstalk between glycolysis and EMT by targeting TGF‐β1, especially in lung adenocarcinoma cells with a highly glycolytic phenotype. The highly glycolytic phenotype of metastatic lung adenocarcinoma cells may account for their increased sensitivity to PFD. Therefore, PFD is a promising drug candidate for NSCLC metabolic therapy, and the metabolic and metastatic phenotypes of NSCLC should be fully considered in the selection of personalized metabolic therapy (Figure 8).

## AUTHOR CONTRIBUTIONS

**Shuling Zhang:** Conceptualization (equal); data curation (equal); funding acquisition (equal); writing – original draft (equal). **Yuanmei Wang:** Data curation (equal). **Daiqin Luo:** Formal analysis (equal); funding acquisition (equal); writing – original draft (equal). **Zhimei Cheng:** Conceptualization (equal); supervision (equal). **Qibing Zeng:** Writing – review and editing (equal). **Guoze Wang:** Methodology (equal); writing – review and editing (equal). **Mengxue Chen:** Resources (equal); visualization (equal). **shuai Zhang:** Supervision (equal); validation (equal); writing – review and editing (equal). **Peng Luo:** Funding acquisition (equal); project administration (equal); resources (equal); supervision (equal); writing – review and editing (equal).

## CONFLICT OF INTEREST STATEMENT

The authors declare no conflict of interest.

## Supporting information


Figure S1.

Figure S2.

Figure S3.

Figure S4.

## Data Availability

All data required to evaluate the conclusions in this paper are appeared in this paper.
