# Supplementary figures and images for: Pirfenidone inhibits TGF‐β1‐induced metabolic reprogramming during epithelial‐mesenchymal transition in non‐small cell lung cancer
Source: J Cell Mol Med. 2023 Dec 23;28(3):e18059. doi: 10.1111/jcmm.18059 (PMC10844763; doi:10.1111/jcmm.18059)

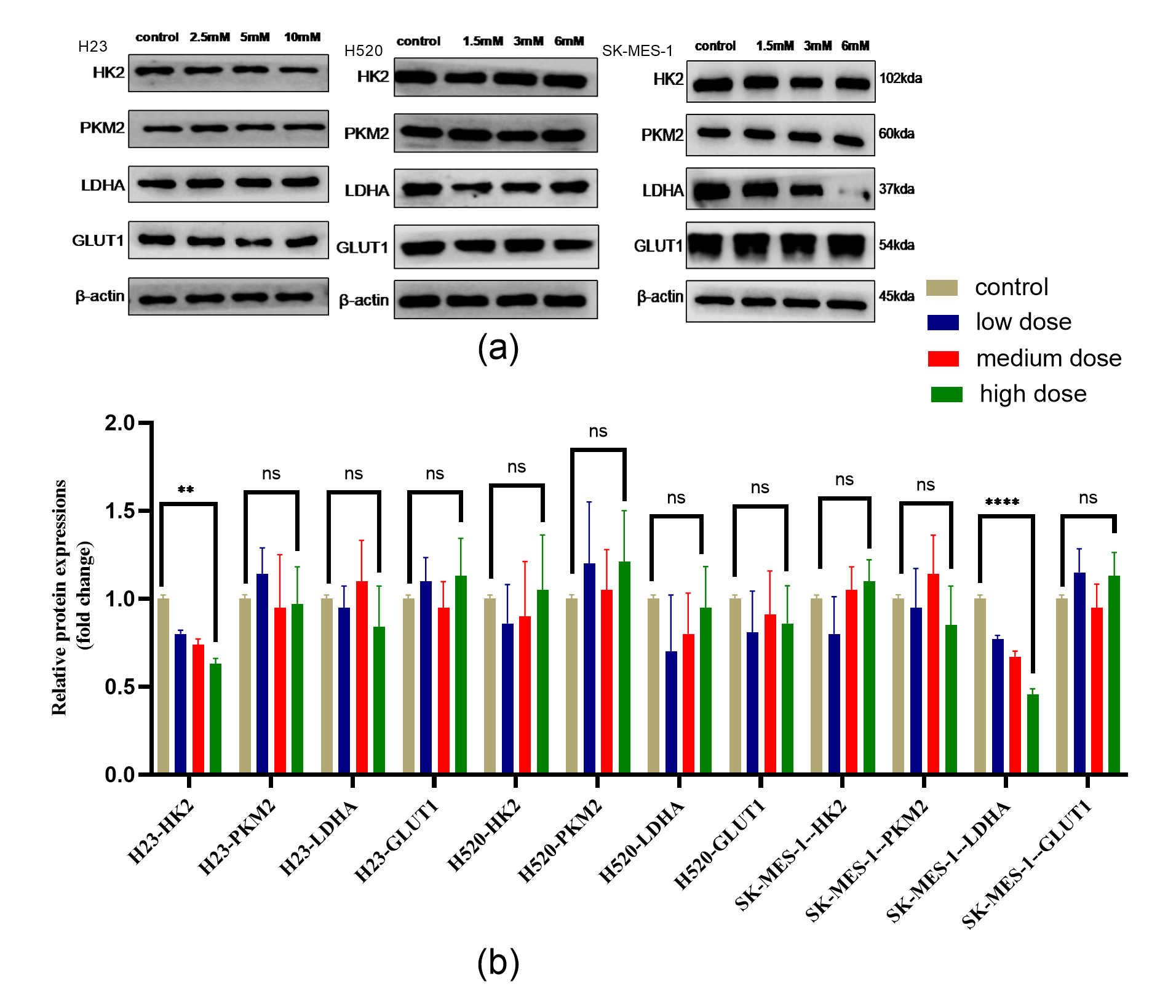

Supplement: Supplementary file 1 — Figure S1. [file JCMM-28-e18059-s002.tif]

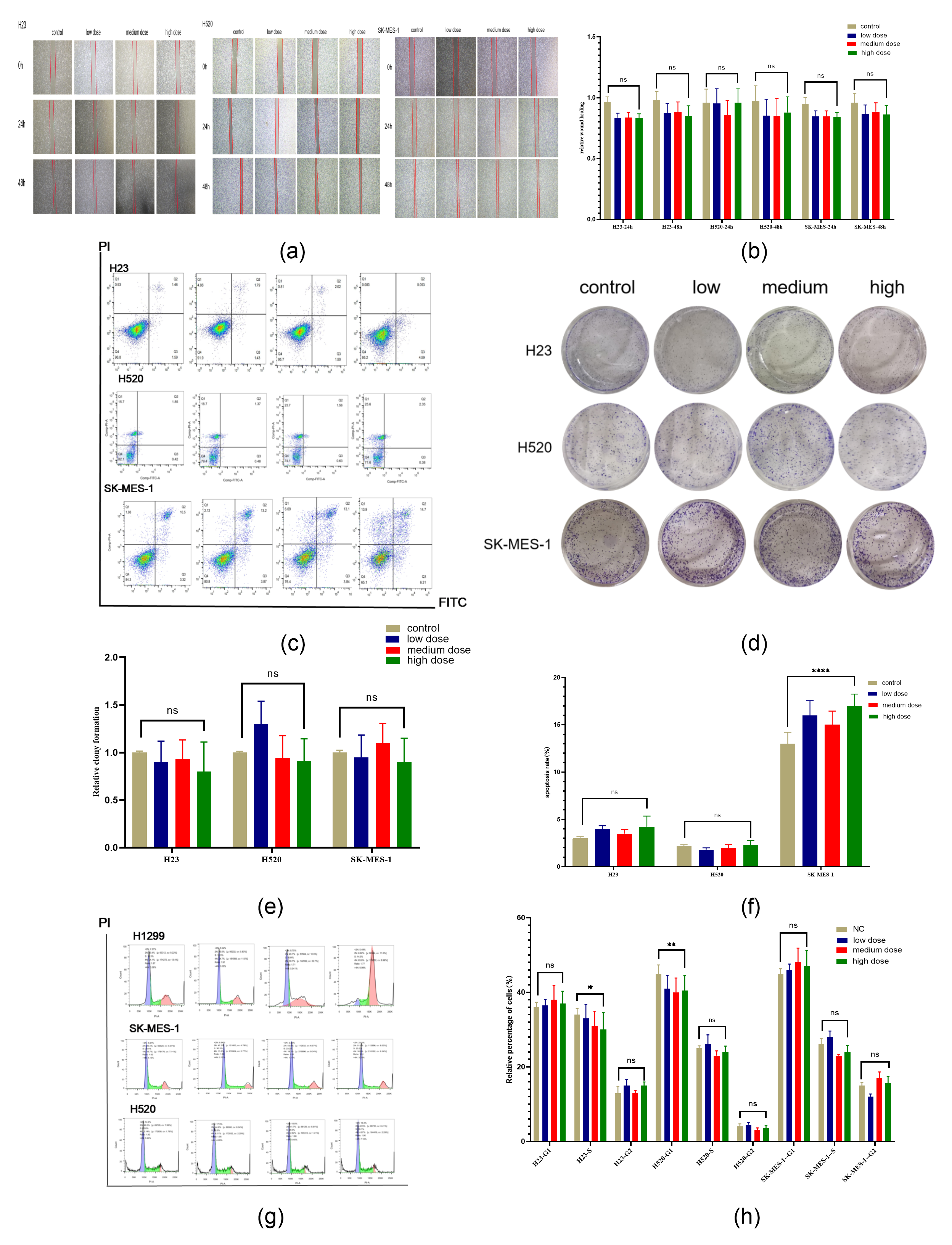

Supplement: Supplementary file 2 — Figure S2. [file JCMM-28-e18059-s003.tif]

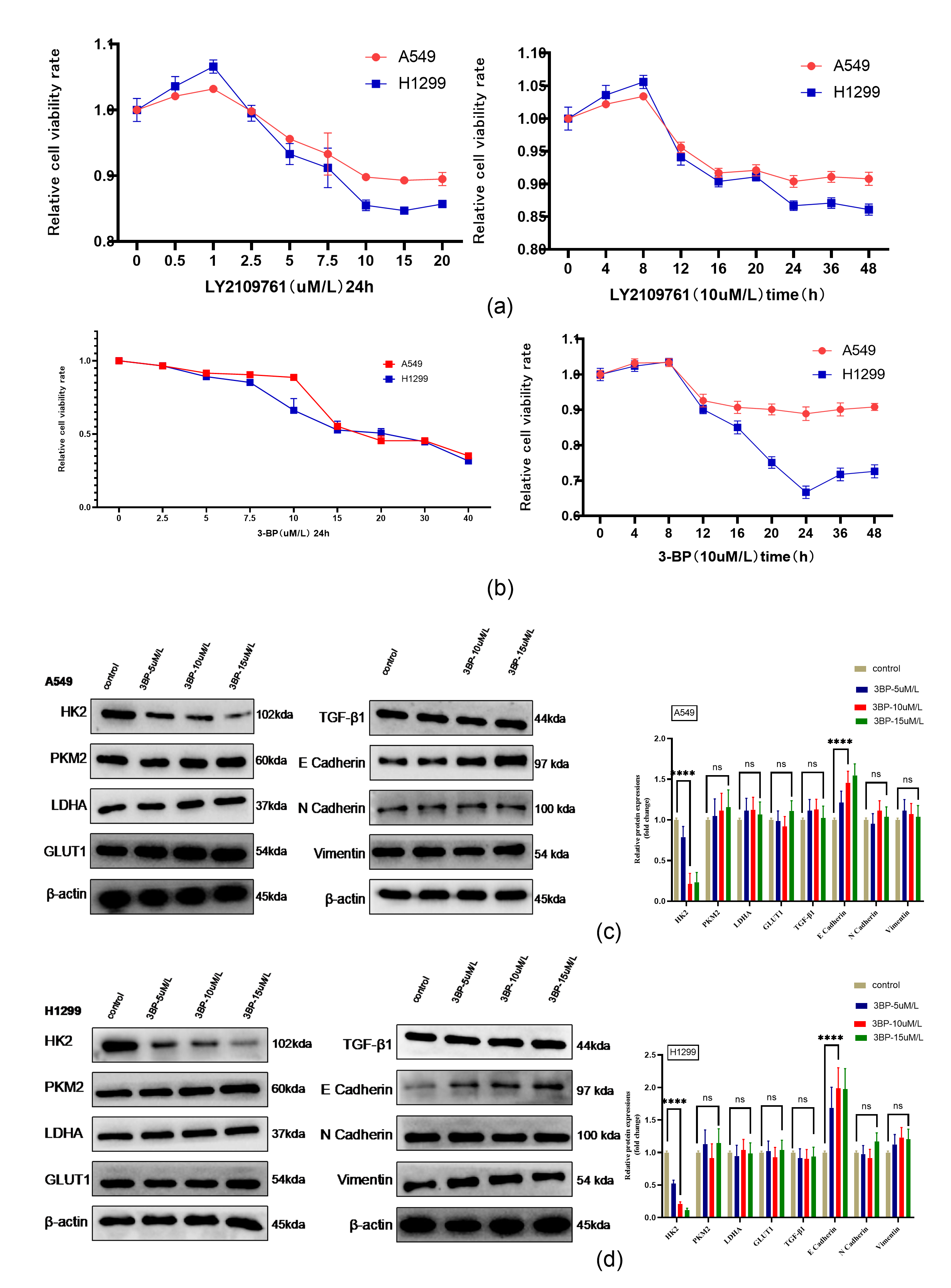

Supplement: Supplementary file 3 — Figure S3. [file JCMM-28-e18059-s004.tif]

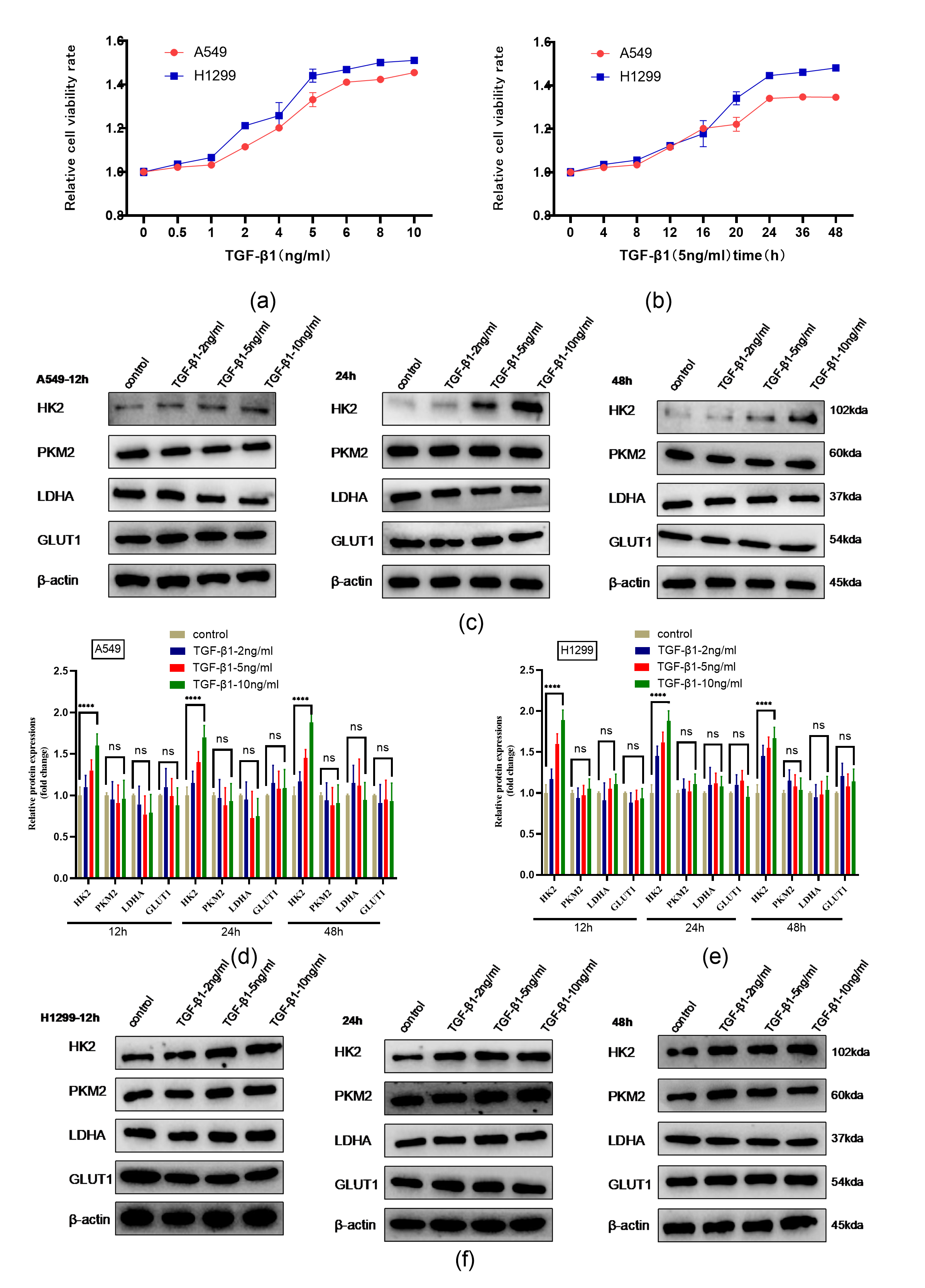

Supplement: Supplementary file 4 — Figure S4. [file JCMM-28-e18059-s001.tif]
